# A Simple Analytical Model for Predicting the Collapsed State of Self-Attractive Semiflexible Polymers

**DOI:** 10.3390/polym8070264

**Published:** 2016-07-16

**Authors:** Wenjun Huang, Ming Huang, Qi Lei, Ronald G. Larson

**Affiliations:** 1 Department of Chemical Engineering, University of Michigan, Ann Arbor, MI 48109-2136, USA; wenjunh@umich.edu (W.H.); huanghun@umich.edu (M.H.); 2 School of Chemical Engineering and Technology, Tianjin University, Tianjin 300072, China; qilei@umich.edu

**Keywords:** semiflexible polymers, Brownian Dynamics simulations, collapsed structures, phase diagram

## Abstract

We develop an analytical model to predict the collapse conformation for a single semiflexible polymer chain in solution, given its length, diameter, stiffness, and self-attractiveness. We construct conformational phase diagrams containing three collapsed states, namely torus, bundle, and globule over a range of dimensionless ratios of the three energy parameters, namely solvent-water surface energy ($\gamma_{s}$), energy of bundle end folds ($\gamma_{e}$), and bending energy per unit length in a torus ($\gamma_{b}$). Our phase diagram captures the general phase behavior of a single long chain (>10 Kuhn lengths) at moderately high (order unity) dimensionless temperature, which is the ratio of thermal energy to the attractive interaction between neighboring monomers. We find that the phase behavior approaches an asymptotic limit when the dimensionless chain length to diameter ratio (L*) exceeds 300. We successfully validate our analytical results with Brownian Dynamics (BD) simulations, using a mapping of the simulation parameters to those used in the phase diagram. We evaluate the effect of three different bending potentials in the range of moderately high dimensionless temperature, a regime not been previously explored by simulations, and find qualitative agreement between the model and simulation results. We, thus, demonstrate that a rather simplified analytical model can be used to qualitatively predict the final collapsed state of a given polymer chain.

## 1. Introduction

Polymer chains collapse into compact globules when the solvent condition changes from good to poor. This “coil-globule transition” is relatively well understood for flexible polymers [1,2]. The globule state minimizes the area of solvent contact of monomers, and makes the collapsed state energetically more favorable than the extended state. However, many polymers exhibit substantial bending stiffness and are considered to be semiflexible, as described by the worm-like chain model [3]. For these chains, a globule state minimizes the surface free energy, but involves substantial bending energy. Therefore, these chains collapse into states that are less dense than the globule, which are overall more thermodynamically favorable. In particular, semiflexible polymer chains, such as biopolymers DNA and (methyl)cellulose, tend to collapse into equilibrium states that balance the bending force that resists the collapse with the attractive force that promotes it. Both experiments [4,5,6] and simulations [7,8,9,10,11,12] have detailed collapse pathways, as well as the conformations of semiflexible polymers, once collapse is complete. A typical pathway for the collapse of a semiflexible polymer chain involves long-lived, partially collapsed, “racquet”-shapes, that are intermediates on the path towards a more compact state, such as a torus, folded bundle, or globule [7,9,10,12].

The ability to predict the collapsed state of a given semiflexible polymer chain based on its structural properties (e.g., diameter, bending stiffness, interaction with solvent, etc.) could be a powerful tool to understand the formation of specific collapsed polymer structures. For example, it was recently shown that methylcellulose gel contains fibrillar structures with a uniform diameter [13]. Such structures are likely to be initiated by the collapse of individual methylcellulose chains when the solution temperature increases [14]. This collapsed state can be qualitatively predicted based on the structural properties of methylcellulose, as explained in a recent simulation study of methylcellulose by Huang et al. [11]. In early work, Schnurr et al. [7] simulated single short stiff chains (2–3 Kuhn steps, $N_{k}$) using bead-spring “pearl necklace” chains and observed the chain evolve from an extended state to various collapsed states. More recently, Seaton et al. [15] reported the phase behavior of simulated 30-mer semiflexible bead-spring “pearl necklace” chains ($N_{k}$ < 10) with various bending stiffnesses over a wide range dimensionless temperatures. With increasing chain stiffness, they observed globules at very small bending stiffness, to bundles of varying aspect ratios at higher bending stiffness, to tori, and to expanded coils at the highest bending stiffness. However, they considered only a single chain length and suggested that the phase diagram could change for much longer chains. In a recent simulation study, Kong et al. [10] detailed various collapsed states and collapse paths exhibited by semiflexible bead-spring polymers at different chain resolutions (i.e., number of beads per Kuhn length), self-attractive strengths, and chain diameters. A conformational phase diagram for polymer chains with a length of five Kuhn steps ($N_{k}$ = 5) as a function of dimensionless self-attraction strength and ratio of chain diameter to Kuhn length was produced. In sum, simulation studies currently available in the literature are limited to bead-spring chains with rather short lengths. However, with the recent advances in computational power, a single chain of length several hundreds of Kuhn steps can now be simulated using standard Brownian Dynamics (BD) simulation model [11]. Therefore, we would like to re-examine the collapsed phase behavior of semiflexible polymer chains over a wider range of chain lengths.

A few analytical models have been developed to characterize the energy of both metastable intermediates during the collapse and the equilibrated conformations of collapsed semiflexible polymers. Schnurr et al. [16] calculated the conformational energy of intermediate “racquet” states and showed that this shape is metastable and that semiflexible self-attractive chains will indeed eventually collapse into energetically more favorable bundles and tori. Their model was derived for low dimensionless temperature (T*, which is the thermal energy $k_{B}T$ divided by the bead-bead attraction energy) where the tori and bundles with “racquet” heads have crystalline packings of the monomer beads used to model the chain. Thus, the model incorporates the packing order of monomers within the filaments, the completeness of the torus, and number of “racquet” heads in a bundle. Moreover, the shape of an ideal “racquet” head is resolved by introducing additional parameters such as total contour length of the chain within the racquet head and the curvature of the racquet. Stukan et al. [17] analyzed the stability of a bundle and a torus formed by single chain using a detailed analytical model, including bead packing energies generated by the number of bead-bead contacts. Their model was derived for collapsed structures of bead spring “pearl necklace” chains at low dimensionless temperature, where the packing order (number of bead-bead contacts) of the filaments in both torus and bundle was considered. For both conformations, they found periodicities in the minimum energy state as a function of chain length due to the periodic completion of windings around the torus, or of parallel filaments in the bundle, respectively. They compared the energy of a torus with perfect hexagonal filament packing to a bundle with ideal tight back folding at the ends, and concluded the torus is always energetically more favorable than the bundle. This is a somewhat striking finding, since bundle structures are generally considered to be stable equilibrium collapsed structures for semiflexible polymer chains and have been observed in both simulations and theoretical studies [10,15,18]. However, it is worth noting that in addition to the limitation on bead spring “pearl necklace” model, the chains Stukan et al. modeled were rather stiff. Therefore, it is reasonable that a bundle with ideal tight back folding at the ends is always energetically more costly to form than a torus. Both the analytical models of Schnurr et al. and of Stukan et al. are applicable to long chains (>100 Kuhn steps).

**Figure 1:**
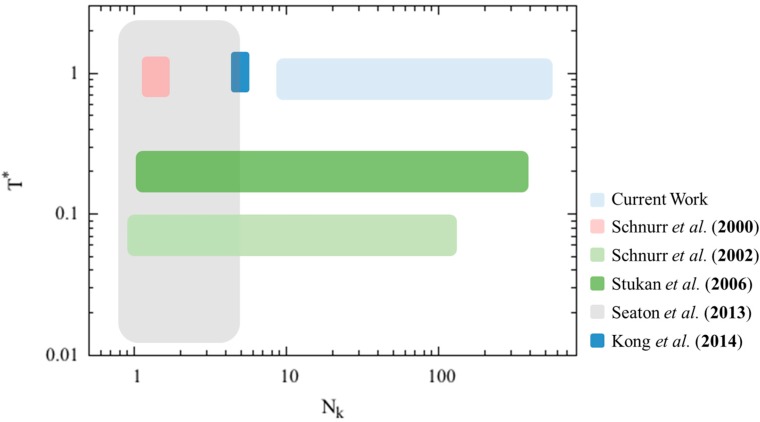
Illustrative phase diagram of the parameter space that has been covered in previous simulations and theoretical models in the literature, and in this work. The *x*-axis is the Kuhn length ($N_{k}$) of the polymer chain, and the *y*-axis is the dimensionless temperature, defined as the *k*_B_*T* divided by the attractive energy per monomer bead used in each study.

The parameter spaces covered by these previous simulation studies, summarized in Figure 1, have mostly focused on high dimensionless temperatures and short chain lengths, and the theoretical modeling studies have resolved the conformational space at low dimensionless temperatures and a wide range of chain lengths. While the hexagonal filament packings in collapsed conformations may be important for short oligomer chains with ideal spherically shaped monomers at low dimensionless temperatures, we are interested in providing qualitative prediction of the final collapsed chain conformation at dimensionless temperature value around 1, similar to the condition in Kong et al.’s work. Therefore, we wish to develop a generic analytical model with less emphasis on the actual filament packing details to predict the minimum free energy conformation of semiflexible polymer chains at high dimensionless temperatures with reasonable accuracy, at least for long polymer chains.

One important consideration in the polymer chain model is the choice of the bending potential. There are two typical bending potentials, namely the harmonic bending potential ($U_{\text{angle},\text{ h}}$) and the cosine bending potential ($U_{\text{angle},\text{ c}}$) (Equation (1a,b)). While Schnurr et al. and Kong et al. chose harmonic potential for their model and Seaton et al. chose cosine potential in their work, Stukan et al. have compared the effect of these two bending potentials on the stability of the torus and bundle. They have found that the choice of the bending potential can lead to different stability of the collapsed structures. For example, for large bending angles, such as the ones in the end fold, the cosine potential is “softer” than the harmonic potential (see Figure S1 at small angle limit), and therefore decreases the overall energy of the end fold. Because Stukan et al. only modeled the chains at low dimensionless temperature with ideal hexagonal packing, we would also like to re-evaluate the effect of the bending potentials on the conformational behavior at higher dimensionless temperature and over a wider range of dimensionless chain length range in this work.

(1a) $$U_{\text{angle},\text{ h}}=\frac{1}{2}K_{\theta }{\left(\theta -\theta_{0}\right)}^{2}$$

(1b) $$U_{\text{angle},\text{c}}=\frac{1}{2}K_{\theta }\left[1-\cos\left(\theta -\theta_{0}\right)\right]$$

(1c) $$U_{\text{angle},\text{s}}=8U_{\text{angle},\text{h}}-14U_{\text{angle},\text{c}}$$

In this work, we develop a much simpler analytical model to predict the formation of torus or bundle states by representing these collapsed states as ideal geometries with specified dimensions, and corresponding surface areas, such as the areas of the folded bundle ends and sides. We then subsume the properties of the chain, namely the number of monomers, monomer diameter, and chain stiffness into surface free energies of both ends and sides, as well as the bending energy per unit length for a chain with a radius of curvature set by the radii of the torus. We then find the geometry of minimum free energy of both torus and bundle for a given set of parameters, and find whether the torus or the bundle has the lower minimum free energy. A phase diagram is produced by recording these minimum free energy conformations. To validate the theoretical derived phase diagram, we also simulate bead-spring “pearl-necklace” chains using Brownian Dynamics (BD) simulations with three different bending potentials. Specifically, in addition to the harmonic and cosine bending potential, we adopt a “stiff” potential ($U_{\text{angle},\text{ s}}$) using a linear combination of the two aforementioned potentials (Equation (1c)) to study the effect of bending potential systematically. We show that the predicted phase diagram agrees with the BD simulation results qualitatively.

The paper is organized as follows. We first discuss the development of the analytical model for the collapsed conformations, and the model we use for the BD simulations. We also discuss how to map the parameters used in the analytical model onto the input parameters for the simulations. We then show the calculated phase diagrams for various dimensionless chain lengths, and compare these phase diagrams with the simulation results.

## 2. Methods

### 2.1. Analytical Model

**Figure 2:**
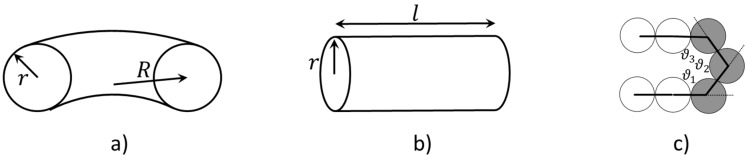
Schematics of torus (**a**) and bundle (**b**); (**c**) Schematic of a generic end fold we consider in this work, which has three exposed monomers (colored in gray). The angles in the end fold, $\vartheta_{1-3}$, are formed among three consecutive monomer beads. For simplicity, these angles are taken to be 120°.

We consider three possible collapsed conformations for a single polymer chain, namely torus (T), bundle (B), and globule (G). The “pearl-necklace” polymer chain is modeled using beads connected by short stiff springs; each chain contains *N* beads of diameter *σ*, and the contour length of the chain is *L*. We introduce energy penalty parameters for exposed monomer surface ($\gamma_{s}$), end folds in the bundle ($\gamma_{e}$), and chain bending in the torus ($\gamma_{b}$), respectively. The former two parameters have units of free energy per unit area, while the latter has units of free energy per unit curvature squared per unit chain length. This yields three dimensionless ratios, namely $\gamma_{b}/\gamma_{e}\sigma^{3}$, $\gamma_{e}/\gamma_{s}$, and $L^{*}\;\equiv L/\sigma$. The other quantities with units of length are also scaled by σ to make them dimensionless. We approximate the stretched chain as a long and thin tube, with the volume of the chain $V_{c}$ is made dimensionless as $V_{c}/\sigma^{3}\;\equiv$ $\pi L^{*}/4$. We focus on the two collapsed conformations, namely the torus and the bundle. For each of these, we estimate the free energy by summing up the contributions from the lateral surface (for the bundle and the torus), and from the areas of the two end caps (for the bundle), and from chain bending (for the torus). We then differentiate with respect to radius r* (≡ r/σ) for each structure (Figure 2) to obtain the lowest free energy state.

In the torus model, there are two dimensionless lengths, namely ${r_{t}}^{*}$ (≡r/σ) and R* (≡R/σ). As shown in Figure 2a, *r* is the radius of the cross section of the torus, and *R* is the distance from the center of the torus to the center of the cross section. We start by equating the volume of the free chain ($V_{c}$) to the volume of the torus ($V_{t}$) (Equation (2a)). We then express the free energy of the torus ($G_{t}$) as the sum of surface energy and bending energy, and derive the expression for dimensionless free energy ${G_{t}}^{*}$. This free energy is made dimensionless with $\gamma_{s}\sigma^{2}$, which is comparable to the free energy of exposure of a single bead to solvent. We perform an analytical differentiation and obtain the value for ${r_{t}}^{*}$ at which the free energy is minimized (note: the second derivative $d^{2}G_{t}^{*}/dr_{t}{*}^{2}$ is always positive) (Equation (2b)). We do not allow R* to be smaller than $2{r_{t}}^{*}$, or the torus will self-intersect and the “donut hole” of the torus will disappear.

(2a) $$\frac{1}{4}\pi L^{*}=\pi {r_{t}}^{*2}2\pi R^{*}\;$$

(2b) $$G_{t}=\gamma_{s}A_{s}+\gamma_{b}\frac{L}{R^{2}}\;\Rightarrow \;{r_{t}}^{*}={\left(\frac{{L^{*}}^{2}}{512\pi \frac{\gamma_{b}}{\gamma_{s}\sigma^{3}}}\right)}^{\frac{1}{5}}$$

In the bundle model, we define the dimensionless radius $r_{b}^{*}$ (≡r/σ) and bundle length l* (≡l/σ), as shown in Figure 2b, where *r* is the radius of the bundle end cap. Again, we equate the volume of the free chain ($V_{c}$) to the volume of the bundle ($V_{b}$) (Equation (3a)). We then express the free energy of the bundle ($G_{b}$) as the sum of lateral surface energy and the end cap energy, and derive the expression for dimensionless ${G_{b}}^{*}$. Note that the surface energy due to the exposed monomers in the end caps is included in the first term. We differentiate the expression and obtain the value of r* at which the free energy is minimized (note: the second derivative $d^{2}G_{b}^{*}/dr_{b}{*}^{2}$ is always positive) (Equation (3b)). We do not allow l* to be smaller than $2{r_{b}}^{*}$ to maintain the ratio of the bundle diameter to its length below unity ($2{r_{b}}^{*}/l^{*}\leq 1$), so that it is truly a “bundle” and not a condensed globule or disk. If, on the other hand, the ratio $2{r_{b}}^{*}/l^{*}$ is greater or equal to 0.5, we consider the structure to be a globule. A detailed derivation for both torus and bundle models are given in the Supplementary Materials.

(3a) $$\frac{1}{4}\pi L^{*}=\pi r_{b}{*}^{2}l^{*}\;$$

(3b) $$G_{b}=\gamma_{s}A_{s}+\gamma_{e}A_{e}\;\Rightarrow \;{r_{b}}^{*}=\frac{1}{2}{\left(\frac{L^{*}}{1+\frac{\gamma_{e}}{\gamma_{s}}}\right)}^{\frac{1}{3}}$$

Defining $k_{1}\equiv \gamma_{e}/\gamma_{s}$, and $k_{2}\equiv \gamma_{b}/\gamma_{e}\sigma^{3}$, we derive the exact solutions for the boundaries between globule and bundle, and between bundle and torus in the asymptotic limit of large L*. A bundle can be considered to be a globule when the ratio $2r_{b}^{*}/l^{*}$ is greater or equal to 0.5 (Equation (4a)). Because the shape of the globule, like that of a bundle, is solely determined by the ratio $\gamma_{e}/\gamma_{s}$, the exact solution to the boundary between globule and bundle is simply the horizontal line at which $\gamma_{e}/\gamma_{s}$ equals 1 (Equation (4c)).

To derive the boundary between the bundle and the globule, we consider the constraint for globule geometry:

(4a) $$0.5=\;\frac{2r_{b}^{*}}{l^{*}}$$

Inserting $l^{*}$ from Equation (3a), $r_{b}^{*}$ from Equation (3d), and $k_{1}\equiv \gamma_{e}/\gamma_{s}$ gives:

(4b) $$0.5=\frac{{\left(\frac{L^{*}}{1+k_{1}}\right)}^{\frac{1}{3}}\;}{\frac{L^{*}}{4{r_{b}^{*}}^{2}}}$$

Inserting $r_{b}^{*}$ from Equation (3d) again and Equation (4b) reduces to Equation (4c), which leads to a trivial solution for $k_{1}$:

(4c) $$0.5=\frac{1}{1+k_{1}};\;\text{thus}\;k_{1}=1$$

Next, we derive the exact solution to the boundary between the torus and bundle phase, obtained by equating their free energies yields a relationship between $k_{2}$ and $k_{1}$ (Equation (5c)). At the limit of large $k_{1}$, Equation (5c) reduces to a simple power law relationship $k_{2}\sim {k_{1}}^{2/3}$ (Equation (6)). We take the two constraints on the torus into consideration, namely a torus has to be non-self-intersecting, and the thickness of the cross section has to be more than one bead, which gives an upper and a lower bound on the $k_{1}$ value in the expression for phase boundary (Equation (5c)), shown in Equation (7a). As a result, $k_{1}$ must exceed 2.63, given by Equation (7c). The upper bound on $k_{1}$, in Equation (7c), is a function of the dimensionless chain length ($L^{*}$).

To derive the boundary between the torus and the bundle, we equate their optimized free energies:

(5a) $$G_{t}^{*}=G_{b}^{*}$$

Using the expression for $G_{t}^{*}$ from Equation (2b) and $G_{b}^{*}$ from Equation (3b) gives:

(5b) $$\frac{1}{2}\pi L^{*}{r_{t}^{*}}^{-1}+k_{1}k_{2}\frac{64\pi^{2}}{L^{*}}{r_{t}^{*}}^{4}=\frac{1}{2}\pi L^{*}{r_{b}^{*}}^{-1}+2\pi {r_{b}^{*}}^{2}+k_{1}2\pi {r_{b}^{*}}^{2}$$

Inserting $r_{t}^{*}$ from Equation (2d) and $r_{b}^{*}$ from Equation (3d), then gives:

$$k_{2}=\alpha^{5}{L^{*}}^{\frac{1}{3}}{\left(8+8k_{1}\right)}^{\frac{3}{5}}{k_{1}}^{-1}$$

where:

(5c) $$\alpha =\frac{\frac{6}{5}}{{\left(512\pi \right)}^{\frac{1}{5}}}$$

Equation (5c) is thus the boundary between torus and bundle phase. For large *k*_1_, Equation (5c) reduces to:

(6) $$k_{2}=32\alpha^{5}{L^{*}}^{\frac{1}{3}}{k_{1}}^{\frac{2}{3}}$$

Next, we consider the constraints on the torus geometry:

(7a) $$\frac{1}{2}\leq {r_{t}}^{*}\leq \frac{R^{*}}{2}$$

Inserting $r_{t}^{*}$ from Equation (2d) and $R^{*}$ from Equation (2a) gives:

(7b) $$\frac{1}{2}\leq \frac{{L^{*}}^{\frac{2}{5}}}{{\left(512\pi \right)}^{\frac{1}{5}}{\left(k_{1}k_{2}\right)}^{\frac{1}{5}}}\leq \frac{{L^{*}}^{\frac{1}{3}}}{{\left(16\pi \right)}^{\frac{1}{3}}}$$

Inserting $k_{2}$ from Equation (5c), we obtain the upper and lower limit for the $k_{1}$ value in Equation (5c), due to the constraints on the torus geometry:

(7c) $$2.63\leq k_{1}\leq 0.58L^{*}-1$$

Because our theory does not consider fluctuations on the scale of the structure (torus, bundle, or globule), and also neglects local bead ordering, the range of temperatures for which our model is valid must be such that the temperature is high relative to the energy of interactions of individual beads, which is of order ε, but must be very low relative to the total interaction energy of the entire molecule, which is of order N ε. For both of these conditions to hold, our model is restricted to long chains (i.e., $L^{*}$ > 100). Because we set the dimensionless temperature (T* = $\;k_{B}T/\varepsilon$) to be unity, our theory is expected to hold at dimensionless temperatures between 1 and << *N*.

### 2.2. Simulation Details

The Brownian Dynamics (BD) simulations in this study were performed using the LAMMPS simulation package [19] (ver. May 2015) in an NVE ensemble. Dimensionless LJ (Lennard-Jones) units were used in the simulations, where the Boltzmann constant (*k*_B_) and the three fundamental units, namely mass (*m*), distance (σ), and energy (ε), were set to be unity. A Langevin thermostat [20] was used in all simulations, with the damping coefficient set to 10, which leads to an overdamped simulation, and reduced temperature (T*) to be unity (*k*_B_T* = 1). The time scale *τ* was defined to be $\sqrt{m\sigma^{2}/\varepsilon }$ , and a typical time step of 0.005*τ* was used. Simulations were carried out for at least $10^{8}$ steps. We tracked the radius of gyration (*R*_g_) to monitor the collapse behavior of the chain. The polymer chain was modeled using beads and stiff springs, with each bead representing a generic monomer in the polymer chain. The CG interaction potential (Equation (1a–c), Equation (8a–c)) included both bonded and non-bonded interactions. Harmonic bond and interactions were used to model the bonded interactions, while the Lennard-Jones 12-6 potential was used to model the non-bonded interaction.

(8a) $$U_{\text{CG}}=\;U_{\text{bond}}+U_{\text{angle}}+U_{\text{non}-\text{bonded}}$$

(8b) $$U_{\text{bond}}=\frac{1}{2}K_{b}\;{\left(l-l_{0}\right)}^{2}$$

(8c) $$U_{\text{non}-\text{bonded}}\left(r\right)=4\varepsilon \left[{\left(\frac{\sigma }{r}\right)}^{12}-{\left(\frac{\sigma }{r}\right)}^{6}\right]\;$$

We considered three bending potentials in the simulations, namely harmonic, cosine, and stiff bending potential (Equation (1a–c)); we have plotted these in Figure S1. By design, the stiff bending potential produces a progressively higher energy penalty than the harmonic bending potential as the bending angle increases. Therefore, given the same generic end fold, such as the one shown in Figure 2c, the cosine potential gives the smallest energy penalty, followed by harmonic and the stiff potential. The bead diameter (σ) in simulation is the same as the bead diameter (σ) in our theory. In the simulations, we hold the interaction strength (ε) and bead diameter (σ) constant at 1.0, and vary the bending potential coefficient ($K_{\theta }$) from 1.5 to 150. The value of persistence length ($l_{P}$) scaled by length unit (σ), when using harmonic potential, is slightly larger than the value of $K_{\theta }$ scaled by energy unit (ε). (i.e., $K_{\theta }/\varepsilon$ of 1.5 gives $l_{P}/\sigma$ roughly 2).

We can map the energy terms used in the analytical model, namely $\gamma_{s}$, $\gamma_{e}$, and $\gamma_{b}$, to the parameters used in the simulations. $\gamma_{s}$ is the free energy penalty a bead pays for being exposed to the solvent. We approximate this free energy by the LJ potential energy of an exposed bead. Beads that interact through an LJ potential in the dense state will pack closely together with numbers of neighbors not too far from 12, that of a face-centered cubic (FCC) with interaction strength of 1ε per contact, which is the depth of the potential well. The contribution per bead in the pair is 0.5ε per contact, and the total potential energy for a non-exposed bead is thus 6ε. Here, we consider an exposed bead loses potential energy contribution from 8 neighboring beads, loosely based on the structure we observed in the simulations. Therefore, $\gamma_{s}$ is approximated to be the potential energy difference (4ε) divided by the cross sectional area of the bead ($\pi \sigma^{2}/4$). $\gamma_{b}$ is the product of bending coefficient ($K_{\theta }$) and bead diameter (σ) (Equation (5a–b)). We consider a generic end fold with three exposed monomer beads (Figure 2c). For simplicity, we take all three angles in the end fold to be 120 degrees. Therefore, $\gamma_{e}$ is the ratio between energy of the fold ($E_{\text{fold}}$), calculated using the three bending potentials respectively, and the approximated exposed end fold surface area of the three monomer beads ($3\sigma^{2}$) (Equation (5c)). The above parameters for mapping are very rough approximations; in general, the structure of the fold and beads packing will depend on the parameters of the model and the dimensionless temperature. However, to maintain the simplicity and generality of the model, we avoid these complexities. The more detailed model of Stukan et al. [17] considered in detail the structure of the fold and beads packing, as reviewed above. Particularly, their model requires re-analysis for each specific end fold conformation, while our simple model should be quite general, although qualitative. In typical simulations, including those discussed here, both end fold and bending energies arise from the same bending potential; the fold is simply an extreme bend, whose energy is set by the same potential, but with smaller radius of curvature. Therefore, the dimensionless ratio $\gamma_{b}/\gamma_{e}\sigma^{3}$ is a constant for a given bending potential. The three bending potentials that we considered allow us to access various $\gamma_{b}/\gamma_{e}\sigma^{3}$ ratios and validate the trend predicted by the theory.

(9a) $$\gamma_{s}=\frac{16\varepsilon }{\pi \sigma^{2}}$$

(9b) $$\gamma_{b}=K_{\theta }d$$

$$\gamma_{e}=\frac{E_{\text{fold}}}{A_{\text{fold}}}=\frac{E_{\text{fold}}}{3\sigma^{2}}$$

(9c) $$E_{\text{fold}}=3U_{\text{angle}}\left(120{}^{\circ}\right)$$

## 3. Results and Discussion

**Figure 3:**
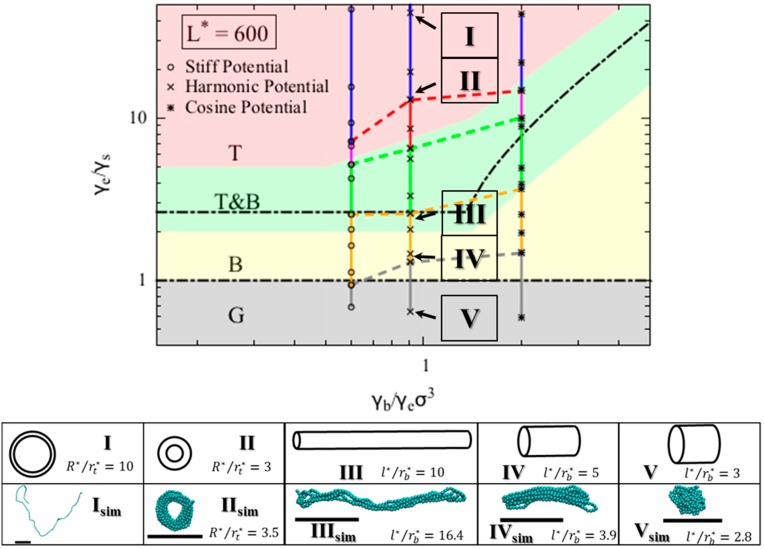
Phase diagrams for polymer chain with dimensionless chain length L* = *L*/σ = 600. Each symbol on the phase diagram marks the parameter values at which a simulation was conducted. Specifically, open circles, crosses, and asterisk symbols are the simulation runs using stiff, harmonic, and cosine bending potentials respectively. A vertical bar connects simulations data producing the same type of structure. The color of the vertical bars, of the dashed lines, and of the regions of the diagram producing this structure coded as follows: blue—random coil (RC), pink—fluctuating between random coil and torus (RC&T), red—torus (T), green—fluctuating between torus and bundle (T&B), yellow—bundle (B), and black- globule (G). Note the pink and blue regions only occur in simulations because we do not model random coil phase. We regard the simulated T&RC phase simply as a torus phase (T) because we do not model the random coil (RC) phase analytically. The dashed lines connecting the vertical bars mark the upper boundaries of the phases obtained from the simulations (e.g., simulation results between yellow and grey dashed lines resulted in a bundle as the final collapsed structure). Exact solutions for the boundaries between the three collapsed phases are shown on the phase diagram (black dash-dotted lines). We have selected representative conformations in each phase, pointed by the arrows. We have calculated the theoretical aspect ratios for these conformations, depicted in the insets I–V. We have also conducted simulations using harmonic bending potential under the same conditions, and the final snapshots from the simulations are supplied in additional insets I–V with subscript “sim”.

Within our model, the collapsed state of a single polymer chain is controlled by three dimensionless quantities, namely $\gamma_{b}/\gamma_{e}\sigma^{3}$, $\gamma_{e}/\gamma_{s}$, and L* =L/σ. A fourth dimensionless parameter, the dimensionless temperature, $k_{B}T/\varepsilon$, influences when the chain will remain a random coil, rather than collapsing, but we consider long chains here at moderate values of $k_{B}T/\varepsilon$, and this parameter therefore does not enter our theory. On the “phase diagram” shown in Figure 3, the y-axis is the ratio of the end fold energy to the surface energy, while the x axis is the ratio of the bending energy to the product of end fold energy and bead diameter cubed. We vary the two dimensionless ratios over a range of values to determine a “phase diagram” of lowest energy conformation. Specifically, for each $\gamma_{e}/\gamma_{s}$ and $\gamma_{b}/\gamma_{e}\sigma^{3}$ pair, we compute the resulting minimized free energies for all three conformations, and record the lowest energy conformation on the phase diagram. The phase diagram for L* = 600 is shown in Figure 3, which includes regions for each of the three phases that we modeled for a polymer chain. The globule (G) conformation is observed when surface energy penalty dominates (small $\gamma_{e}/\gamma_{s}$ values; see inset V in Figure 3). Chains in this region can be regarded as flexible. The chain adopts either torus or bundle configuration when it has moderate bending and end fold energy penalties. Specifically, when the end surface energy penalty is high but the bending energy penalty is low, the polymer chain collapses into a torus (T). The aspect ratio of this torus (R*/r*) increases as the bending energy increases; the cross section of the torus becomes thinner and its radius increases, which helps the chain to reduce the high bending energy penalty. This can be seen from the insets I and II in Figure 3. On the other hand, when the end fold energy penalty is low but bending energy penalty is high, a bundle (B) is formed. The aspect ratio of the bundle (l*/r*) increases as the end fold energy term increases; the bundle becomes thinner and longer, which helps the chain to reduce the end fold energy at an expense of having more beads exposed along its side. Schematics of bundles with different aspect ratios are in the insets III and IV in Figure 3. We mark the exact solutions to the boundaries between collapsed phases, derived in the theory section on Figure 3. The boundary between globule and bundle (Equation (4c)) is a horizontal line at $\gamma_{e}/\gamma_{s}=1$. We note that the upper bound of the boundary between torus and bundle that we present in Equation (7c), namely $\gamma_{e}/\gamma_{s}\leq 0.58L^{*}-1$, is not visible, because the value of $\gamma_{e}/\gamma_{s}$ at the upper bound exceeds the x-axis range. For a small $\gamma_{b}/\gamma_{e}\sigma^{3}$ (*x*-axis value), even though the minimum-free-energy torus is self-intersecting at the given $\gamma_{e}/\gamma_{s}$ and $\gamma_{b}/\gamma_{e}\sigma^{3}$ pair, a torus with minimum possible R*/r* value of 2 can still form and its free energy remains lower than that of a bundle. This results in the horizontal boundary between bundle and torus phase on the phase diagram at small $\gamma_{b}/\gamma_{e}\sigma^{3}$ values. From the simulation trajectories, we observed conformations that fluctuate between two collapsed states, namely torus and bundle. We therefore assume that a chain can fluctuate between two states (i.e., T&B) if the calculated free energy of one state is less than 5% different from that of the other state, and add a transition phase (T&B) on the theoretical phase diagram. The resulting phase diagram captures both the predictions based on theory and the observations from the simulations. Note we expect to see a chain adopting a random coil (RC) conformation when both end fold energy and bending energy penalties dominate (large $\gamma_{e}/\gamma_{s}$ and $\gamma_{b}/\gamma_{e}\sigma^{3}$ values), despite its large solvent-exposed surface area. However, because it is challenging to model the entropic contribution in the free energy of the random coil state to a reasonable accuracy, we are not presenting an analytical model for this state in the current work.

We compare the collapsed conformations predicted by the theory with the ones obtained from the simulations. As we noted earlier, because the bending and the end fold are modeled using the same energy potential, the ratio $\gamma_{b}/\gamma_{e}\sigma^{3}$ is constant for a given bending potential in our simulations. We can map the $K_{\theta }$ and ε values onto the phase diagram using Equation (9a–c), given a generic end fold (Figure 2c). From the simulations that use the harmonic bending potential, we observe various collapsed states as $\gamma_{e}/\gamma_{s}$ value increases (Figure 3 inset *I*_sim_ through *V*_sim_). Specifically, for $\gamma_{e}/\gamma_{s}\;$ values less than 1.3, we observe a globule, and for $\gamma_{e}/\gamma_{s}$ between 1.3 and 2.7, we observe bundles with various aspect ratios. For $\gamma_{e}/\gamma_{s}$ greater than 6.5, tori with various aspect ratios form. In between 2.7 and 6.5, we observe fluctuations between bundle and torus. As expected, simulations show random coils form at large $\gamma_{e}/\gamma_{s}$, here found to be a value greater than 13.

The theoretical diagram is in agreement with the simulation results, and is insensitive to L* for L* ≥ 300. The conformations and their corresponding aspect ratios from both theory and simulation using harmonic bending potential under various conditions are presented in Table 1. The aspect ratios, l*/r* and R*/r* of the simulated conformation were measured by both visual inspection and an in-house analysis code and agree with the theoretically predicted phases for collapsed states, at least at L* = 600. We indeed observed both torus and bundle formed in our simulations within the region predicted by the theory. The aspect ratios of the conformations, however, agree only qualitatively. Nevertheless, the aspect ratios of bundles and tori increase with the bending potential coefficient $K_{\theta }$, as predicted.

We compared the simulation results obtained using different bending potentials. The simulations show transitions from collapsed states to random coil state for all three bending potentials. The transitions from bundle (B) to fluctuation between bundle and torus (T&B) occur at very similar $\gamma_{e}/\gamma_{s}$ values for simulations using stiff and harmonic potentials, and at a higher $\gamma_{e}/\gamma_{s}$ value for simulations using the cosine potential. This trend is in agreement with theoretical prediction. The transition from fluctuation between bundle and torus (T&B) to torus (T) occurs at a higher $\gamma_{e}/\gamma_{s}$ value as $\gamma_{b}/\gamma_{e}\sigma^{3}$ increases. This shows that a chain modeled with a “softer” bending potential that grows less steeply with increasing bending angle is more likely to form a bundle with large aspect ratio. We have marked the upper boundaries of the phases obtained from the simulation results in Figure 3 (i.e., chain in the simulation with mapped $\gamma_{e}/\gamma_{s}$ value between yellow and grey dashed line will result in a bundle as the final collapsed structure). Because we do not model the random coil phase analytically, we simply regard the phase where chain fluctuates between torus and random coil (T&RC) as the torus phase (T). These boundaries obtained from simulations agree qualitatively with the ones predicted by the theory. We note that a globule is merely a very short bundle; therefore, it is often hard to distinguish between a short bundle and a globule from the simulation results, contributing to the deviation on the boundaries between globule and bundle obtained from simulation and theory.

**Figure 4:**
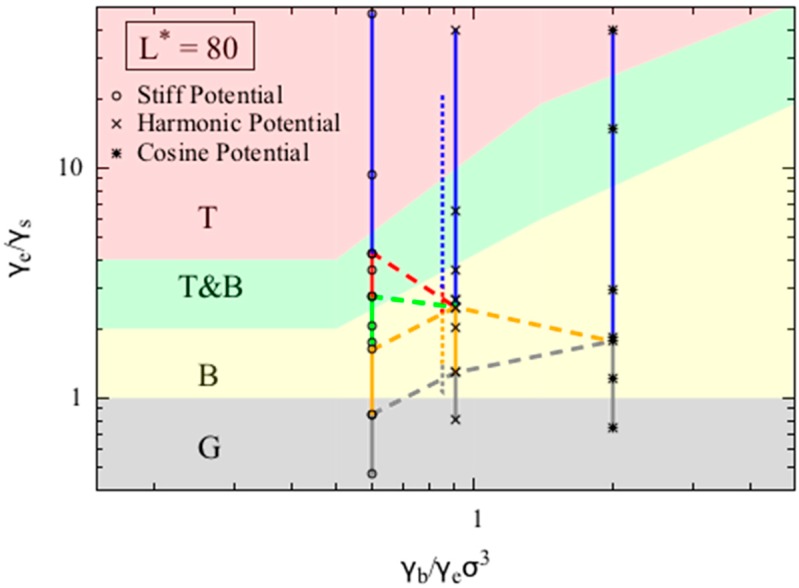
Phase diagrams for polymer chains with dimensionless chain lengths L* of 80. We overlay the simulation results from this study (solid lines) onto all three phase diagrams and the upper boundaries of the phases obtained from the simulations (colored dashed lines) onto the phase diagrams. The color coding and the definitions for the solid and dashed lines are the same as in Figure 3. We also overlay the simulation results from Kong et al. [10] onto the phase diagram (dotted line).

Figure 4 shows that the phase diagrams for dimensionless chain lengths $L^{*}\;$ = 80. For small dimensionless chain lengths L* (i.e., 80), the phase diagram is more sensitive to chain length, and the model becomes less accurate. In Figure 4 we assess the accuracy of our model for $L^{*}\;$ = 80, which was chosen to match the value used in the phase behavior reported by Kong et al. [10] for chains with dimensionless bead diameter $\sigma^{*}$ (defined as the ratio of bead diameter to Kuhn length) of 1/16 and five Kuhn lengths long. Kong et al. adopted a harmonic bending potential in their study. To match the conditions reported in Kong et al. we use the harmonic bending potential form, and set the bead diameter to be 1, the chain length to be 80. Unlike other simulations we report in this work, we keep the bending potential strength ($K_{\theta }$) to be 7.5, corresponding to persistence length (which is half the Kuhn length) to be around 8σ, and then vary the non-bonded interaction strength (ε) to obtain various final equilibrium conformations. We overlay the simulation results from both Kong’s work and our simulations results in Figure 4. Note that because the both models use harmonic bending potentials to model the bending of polymer chains, the two data sets, when mapped onto the phase diagram (Equation (9a–c)), share the same *x*-axis value, and are slightly separated along this axis for clarity only. The two data sets agree well, and the offset along the y-axis is likely caused by the small error in the persistence length estimation.

We also conducted simulations using the other two potentials. Chains with L* = 80 are more likely to form a random coil (RC) when the $\gamma_{b}/\gamma_{e}\sigma^{3}$ ratio increases, as shown by the smaller value of $\gamma_{e}/\gamma_{s}$ at which the random coil state appears. Interestingly, we do not observe either a bundle or a torus for simulations using the cosine bending potential, as the chain transitions directly from the globule state to the random coil state. On the other hand, when using the stiff bending potential, we observe a larger region of torus phase compared to the simulations using harmonic bending potential, which agrees with the theoretical prediction qualitatively. For L* = 80, although the transition between globule (G) and bundle (B) phase in the simulation is predicted relatively well by the simple theory, the transition from bundle (B) to torus (T) clearly deviates from the prediction. Specifically, in the simulations using the harmonic bending potential, the transition from bundles to tori occurs at $\gamma_{e}/\gamma_{s}\;$ = 2.5, while the model predicts that these transitions occur at around $\gamma_{e}/\gamma_{s}$ = 10. This large deviation is expected as our simple continuous model is likely to break down for short chains, for which numbers of folds in the folded state and number of chain wrappings in the torus become few, leading to local minima and maxima in free energy as quantization effects set in. Such effects can indeed by seen in the more detailed models of Schnurr et al. [16] and Stukan et al. [15]. We also tested our model for chains with dimensionless chain length L* = 300 and 1200, and found very similar agreement between theory and simulation as we observed for L* = 600.

## 4. Conclusions

We have developed a simplified continuous analytical model to predict the collapse conformation of a single self-attractive semiflexible polymer chain in solution. We produced from this theory phase diagrams at various dimensionless chain lengths ($L^{*}$) by varying the ratios between three energy parameters, namely the solvent-water surface energy ($\gamma_{s}$), the energy of bundle end fold ($\gamma_{e}$), and the bending energy per unit length in a torus ($\gamma_{b}$). Three phases were modelled in this work—torus, bundle, and globule. We showed that at long chain lengths, L* = 300 or higher, our model predicts the transitions between globules and bundles, and between bundles and tori with reasonable accuracy as shown by Brownian Dynamics simulation results using three different bending potentials. We derived the exact solutions to the boundaries between globule, bundle, and torus phase. However, our model breaks down for short dimensionless chain lengths ($L^{*}$ < 100), for which the detailed packing of the filament becomes more important. Nevertheless, given the good qualitative agreement between theoretical and simulated results at long chain length for transitions between collapsed states, this simplified analytical model may be useful for obtaining quick estimates of the collapsed state of a given polymer chain, and for gaining insight into the factors controlling transitions between collapsed states.

## Figures and Tables

**Table 1:** Comparison of conformations and their corresponding aspect ratios between simulation using harmonic bending potential and theory for chain with L* = 600. Each simulation is repeated three times and the error bar is taken as the standard deviation among the three aspect ratios. The aspect ratios l*/r* and R*/r* are only shown for the bundle and torus conformations.

$K_{\theta }$	ε	$\gamma_{b}/\gamma_{e}\sigma^{3}$	$\gamma_{e}/\gamma_{s}$	Config. (Sim.)	Asp. ratio (Sim.)	Config. (Theory)	Asp. ratio (Theory)
1.5	1	0.65	0.91	G		G	
3.0	1	0.65	1.30	G		G	
7.5	1	0.65	3.23	B	4.3 ± 1.4	B	6.0
10.0	1	0.65	2.77	T&B		T&B	
13.0	1	0.65	4.31	T&B		T&B	
15.0	1	0.65	6.46	T	4.4 ± 1.1	T	2.6
30.0	1	0.65	12.92	T	9.2 ± 1.2	T	4.2
150.0	1	0.65	44.60	RC		T	10.0

## Supplementary Materials

Supplementary Materials can be found at www.mdpi.com/2073-4360/8/7/264/s1.
