# Supplementary material for: A Simple Analytical Model for Predicting the Collapsed State of Self-Attractive Semiflexible Polymers
Source: Polymers (Basel). 2016 Jul 16;8(7):264. doi: 10.3390/polym8070264 (PMC6432155; doi:10.3390/polym8070264)
Supplement: Supplementary file 1 [file polymers-08-00264-s001.pdf]

Supplementary Materials: A Simple Analytical Model for Predicting the Collapsed State of Self-Attractive Semiflexible Polymers

Wenjun Huang, Ming Huang, Qi Lei and Ronald G. Larson

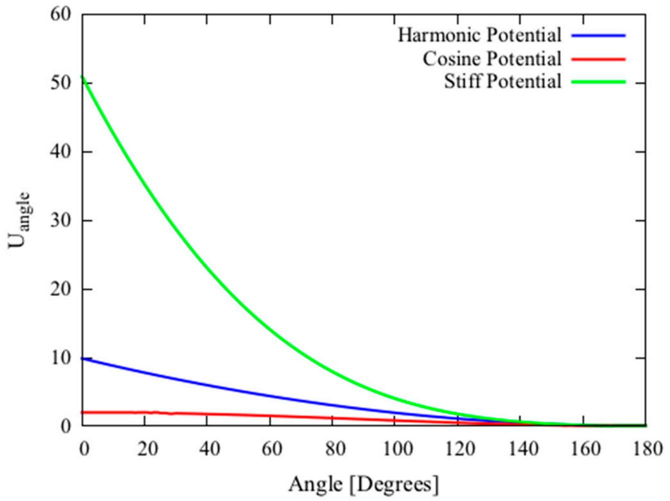

Figure S1. Plot of the three bending potentials considered in the Brownian Dynamics (BD) simulations conducted in this study. The "stiff" potential is a linear combination of the harmonic and cosine bending potentials, given in Equation (1c). This new potential is designed to give larger end fold energy compared to the other two. Using the convention in our BD simulation, the equilibrium angle is set to be 180°.

Here, we provide detailed derivation for torus and bundle model, respectively.
To derive the dimensionless free energy for the torus, we first equate the volume of a torus to that of a stretched chain and obtain the expression for dimensionless radius of the torus (R^*):

$$\frac{1}{4}\pi L^* = \pi {r_t^*}^2 2\pi R^* \implies R^* = \frac{L^*}{8\pi {r_t^*}^2} \tag{S1a}$$

We then express the free energy of the torus (G_t) as the sum of surface energy and bending energy:

$$G_t = \gamma_s A_s + \gamma_b \frac{L}{R^2} = \gamma_s (2\pi r_t 2\pi R) + \gamma_b \frac{L}{R^2} \tag{S1b}$$

We then scale this free energy by $\gamma_s d^2$ and all lengths (L, r_t, R) by d to make these quantities dimensionless. Inserting R^* from Equation (S1a) then gives:

$$G_t^* = \frac{G_t}{\gamma_s \sigma^2} = \frac{1}{2}\pi L^* {r_t^*}^{-1} + \frac{\gamma_b}{\gamma_s \sigma^3}\frac{64\pi^2}{L^*}{r_t^*}^4 \tag{S1c}$$

Setting the first derivative ($dG_t^*/d{r_t}^*$) equal to 0 and solving for ${r_t}^*$ gives the final result:

$$r_t^* = \left(\frac{{L^*}^2}{512\pi \frac{\gamma_b}{\gamma_s \sigma^3}}\right)^{\frac{1}{5}} \tag{S1d}$$

To derive the dimensionless free energy for the bundle, we first equate the volume of a stretched chain to that of a bundle and obtain the expression for dimensionless length of the bundle (l^*):

$$\frac{1}{4}\pi L^{*} = \pi {r_{\mathrm{b}}}^{*2} l^{*} \;\Longrightarrow\; l^{*} = \frac{L^{*}}{4{r_{\mathrm{b}}}^{*2}} \tag{S2a}$$

We then express the free energy of the torus (G_{b}) as the sum of lateral surface energy and the end cap energy:

$$G_{\mathrm{b}} = \gamma_{\mathrm{s}} A_{\mathrm{s}} + \gamma_{\mathrm{e}} A_{\mathrm{e}} = \gamma_{\mathrm{s}}(2\pi r_{\mathrm{b}} l + 2\pi {r_{\mathrm{b}}}^{2}) + \gamma_{\mathrm{e}} 2\pi {r_{\mathrm{b}}}^{2} \tag{S2b}$$

We normalize the free energy by $\gamma_{\mathrm{s}} d^{2}$ and all length terms (l, r_{b}) by d to make these quantities dimensionless. Inserting l^{*} from Equation (S2a), gives:

$$G_{\mathrm{b}}^{*} = \frac{G_{\mathrm{b}}}{\gamma_{\mathrm{s}}\sigma^{2}} = \frac{1}{2}\pi L^{*} {r_{\mathrm{b}}}^{*-1} + 2\pi {r_{\mathrm{b}}}^{*2} + \frac{\gamma_{\mathrm{e}}}{\gamma_{\mathrm{s}}} 2\pi {r_{\mathrm{b}}}^{*2} \tag{S2c}$$

Setting the first derivative ($dG_{\mathrm{b}}^{*}/d{r_{\mathrm{b}}}^{*}$) equals to 0 and solving for ${r_{\mathrm{b}}}^{*}$ gives:

$${r_{\mathrm{b}}}^{*} = \frac{1}{2}\left(\frac{L^{*}}{1 + \frac{\gamma_{\mathrm{e}}}{\gamma_{\mathrm{s}}}}\right)^{\frac{1}{3}} \tag{S2d}$$

© 2016 by the authors; licensee MDPI, Basel, Switzerland. This article is an open access article distributed under the terms and conditions of the Creative Commons Attribution (CC-BY) license (http://creativecommons.org/licenses/by/4.0/).
